# Chemoecological Screening Reveals High Bioactivity in Diverse Culturable Portuguese Marine Cyanobacteria

**DOI:** 10.3390/md11041316

**Published:** 2013-04-22

**Authors:** Pedro N. Leão, Vitor Ramos, Patrício B. Gonçalves, Flávia Viana, Olga M. Lage, William H. Gerwick, Vitor M. Vasconcelos

**Affiliations:** 1 CIMAR/CIIMAR—Interdisciplinary Centre of Marine and Environmental Research, University of Porto, Rua dos Bragas 177, Porto 4050-123, Portugal; E-Mails: vtr.rms@gmail.com (V.R.); patriciojbgoncalves@hotmail.com (P.B.G.); vmvascon@fc.up.pt (V.M.V.); 2 Department of Biology, Faculty of Sciences, University of Porto, Rua do Campo Alegre, Porto 4169-007, Portugal; E-Mails: flavia.viana@biology.au.dk (F.V.); olga.lage@fc.up.pt (O.M.L.); 3 Scripps Institution of Oceanography, University of California, San Diego, La Jolla, CA 92093, USA; E-Mail: wgerwick@ucsd.edu; 4 Skaggs School of Pharmacy and Pharmaceutical Sciences, University of California, San Diego, La Jolla, CA 92093, USA

**Keywords:** cyanobacteria, chemical ecology, screening, bioactive compounds, secondary metabolites, phylogenetic analysis

## Abstract

Marine cyanobacteria, notably those from tropical regions, are a rich source of bioactive secondary metabolites. Tropical marine cyanobacteria often grow to high densities in the environment, allowing direct isolation of many secondary metabolites from field-collected material. However, in temperate environments culturing is usually required to produce enough biomass for investigations of their chemical constituents. In this work, we cultured a selection of novel and diverse cyanobacteria isolated from the Portuguese coast, and tested their organic extracts in a series of ecologically-relevant bioassays. The majority of the extracts showed activity in at least one of the bioassays, all of which were run in very small scale. Phylogenetically related isolates exhibited different activity profiles, highlighting the value of microdiversity for bioprospection studies. Furthermore, LC-MS analyses of selected active extracts suggested the presence of previously unidentified secondary metabolites. Overall, the screening strategy employed here, in which previously untapped cyanobacterial diversity was combined with multiple bioassays, proved to be a successful strategy and allowed the selection of several strains for further investigations based on their bioactivity profiles.

## 1. Introduction

Marine cyanobacteria are known to produce a diverse array of secondary metabolites, many of which possess potent biological activities [1]. While novel structures are frequently reported from these sources, a large number of cultured and uncultured taxa have yet to be studied regarding their constituents; our knowledge of the chemical diversity present in these organisms is still quite limited.

Among marine cyanobacteria, the most prolific sources for the discovery of bioactive secondary metabolites have been tropical species, in particular *Moorea* (formerly *Lyngbya* [2]) species and other members of the order Oscillatoriales [1,3,4]. A common argument is that, analogously to what is observed in the plant kingdom, tropical regions are of higher biodiversity, and thus the fierce competition for space markedly drives secondary metabolite evolution and chemotype diversification (e.g, [5]). Nevertheless, there may be other factors as well that explain the higher number of compounds being reported from tropical cyanobacteria. In particular, a large fraction of the reported marine cyanobacterial secondary metabolites have been discovered by only a few research groups (e.g., R. Moore and W. Gerwick laboratories [1,3]), who devoted a large sampling effort to tropical marine regions. Also of importance, tropical benthic cyanobacteria tend to grow to high densities in the environment, forming extensive mats or tufts that are more easily collected by snorkeling or SCUBA diving. The cyanobacterial biomass in these environmental samples is usually sufficient to allow for direct chemical investigations. Temperate areas of the globe (and even polar regions) harbor a considerable diversity of marine cyanobacteria [6,7], but very little is known regarding their secondary metabolism. Cyanobacteria in these marine environments are seldom found in large mats or tufts (the few exceptions are likely to be found in flat beaches, under low wave energy conditions), thus the body of knowledge on their secondary metabolites has been almost exclusively derived from biomass obtained from laboratory cultures (e.g., [8]). One notable exception has been the discovery of the cyclic peptide nodularin from *Nodularia* *spumigena* bloom material from the brackish Baltic Sea [9]. It is thus plausible that the perceived relative richness in natural products by marine tropical cyanobacteria is somewhat overestimated.

Two distinct and sometimes complementary strategies have facilitated the discovery of marine cyanobacterial natural products. Isolation of metabolites based on distinctive NMR signatures (or NMR-guided isolation) has been a successful strategy in the discovery of relatively abundant compounds. However, bioassay-guided isolation is perhaps the most successful approach to identify the active components from extracts of these organisms, sometimes present only in minute amounts. The outcome of this strategy is biased by the choice of the bioassay, which inevitably influences the isolation process, and may overlook metabolites with interesting chemical structures or bioactivities other than those used to guide the isolation process. In addition, a large proportion of the bioassays employed in discovery programs with marine cyanobacteria are application-related (for example, cancer cell line cytotoxicity assays, or antibacterial assays using clinically-relevant strains). While this approach has yielded and continues to yield promising results (e.g., largazole, see Hong and Luesch [10] and the carmaphycins, see Pereira *et al.* [11]), the assays are usually not directly connected to the natural roles that cyanobacterial metabolites may play, and thus, many bioactive metabolites may never be investigated for this reason [12].

With these premises in mind, in the current study we conducted a screening investigation with the ultimate goal of selecting promising marine strains of cyanobacteria for the isolation of new chemical entities. We utilized thirteen strains of laboratory isolated and cultured marine cyanobacteria obtained from the intertidal zones of rocky beaches in Portugal. The phylogenetic diversity of some of these strains has been recently studied [13], and they represent an untapped and renewable source of interesting new metabolites. These were evaluated using a bioassay strategy that was designed to be more relevant to their putative endogenous function. In this regard, we hypothesized that an increased hit number would be obtained using (a) a larger number of biological assays when compared to traditional approaches which typically use only one or two screening assays, and (b) ecological-related bioassays as opposed to application-related ones.

Our results confirmed our hypothesis that a large percentage of the crude extracts and fractions from the strains would exhibit activity in one or more of the ecologically-relevant bioassays. These assay results can be used to guide future isolation efforts of the active constituents. MS-based dereplication along with a commercial database of marine natural products indicated that active fractions contained, among their more abundant constituents (as estimated by LC-MS profiling), previously unreported masses, supporting the potential of the present approach to discover new cyanobacterial metabolites. This potential also became evident from the bioassay-guided purification of active constituents from one of the strains, *Romeria* sp. LEGE 06013, which led to a glycolipid rich fraction that also contained previously unreported masses. In addition, the diversity of cyanobacteria herein tested allowed comparing bioactivity patterns with phylogenetic relatedness.

## 2. Results

### 2.1. Diversity

A selection of thirteen strains isolated from rocky beaches in Portugal was used in the present study (Table 1).

**Figure 1:**
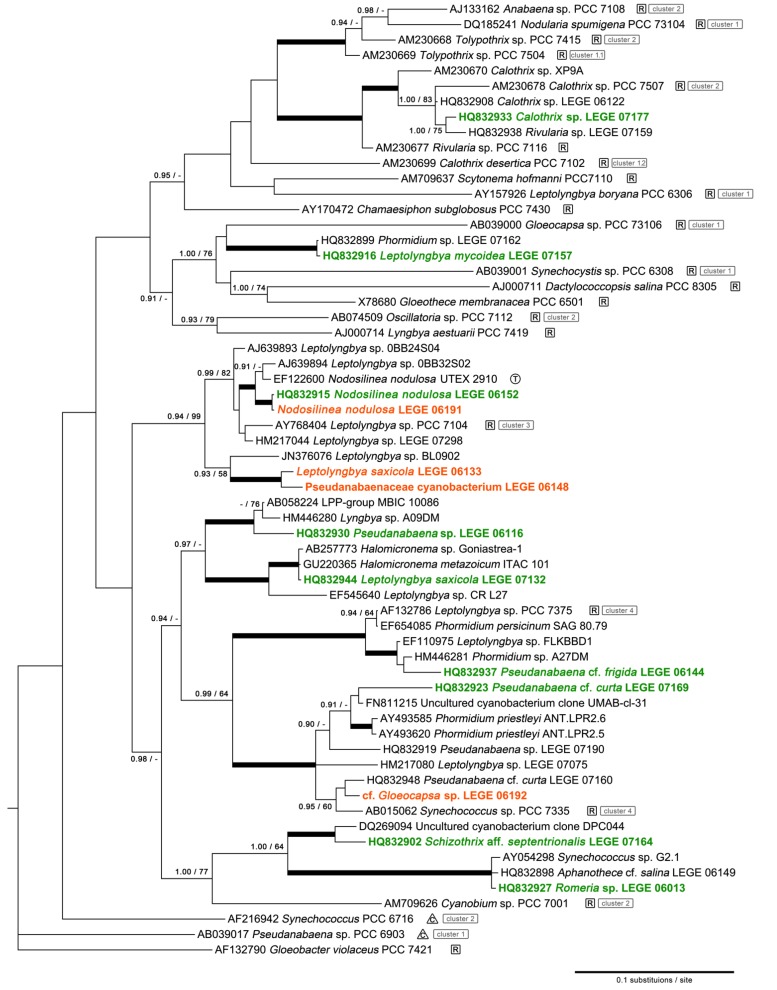
Phylogenetic tree of cyanobacterial 16S rRNA gene sequences illustrating the placement of selected isolates among the cyanobacterial diversity. Topology was obtained by Bayesian inference (BI) (−lnL = 12,819.98). The nodal support values indicated near internal branches were determined by BI and ML methods, respectively; bootstrap values (for ML) below 60% and posterior probability values (for BI) below 0.90 were omitted. Thick lines indicate simultaneous ≥0.95 posterior probability and ≥85% bootstrap values support for tree branches. Reference strains and/or their close relatives are marked with “T” or “C” respectively, while type species (see text for distinction) are marked with “R”. The tree was rooted using *Chloroflexus aurantiacus* J-10-fl (D38365) as an outgroup, which was removed for clarity.

From our phylogenetic analysis (Figure 1), it becomes evident that the selected strains are sufficiently distant in evolutionary terms to provide a heterogeneous set of cyanobacterial diversity. Exceptions are the strains *Nodosilinea nodulosa* LEGE 06152 and LEGE 06191, which are phylogenetically very close and the Pseudanabaenaceae cyanobacterium LEGE 06148 and *Leptlolyngbya* sp. LEGE 06133 which belong to the same sub-cluster. As expectable, *Calothrix* sp. LEGE 07177 is placed within the heterocystous filamentous clade. The filamentous non-heterocystous forms are spread out along the tree, in different clades and sub-clades. *Leptolyngbya saxicola* LEGE 07132 forms a clade with other thin filamentous cyanobacteria, two of them identified as *Halomicronema* spp., but this group is placed distantly from the type species *Halomicronema* sp. TFEP1 [14] (data not shown). The sole unicellular cyanobacterial strain used in this study is situated in a large clade comprising above all filamentous non-heterocystous members of the LPP-group B (Rippka *et al.* [15]) and *Pseudanabaena* spp. from our culture collection. This clade represent the “marine *Leptolyngbya* lineage” established in the Bergey’s Manual of Systematic Bacteriology and is also known to include the free-living unicellular *Synechococcus* sp. PCC 7335 [16,17]. Notwithstanding, the colonial cyanobacterium cf. *Gloeocapsa* sp. LEGE 06192 (see Table S1 and Figure S1 for details) clustered in a subclade with *Synechococcus* sp. PCC 7335 and the filamentous non-heterocystous *Pseudanabaena* cf. *curta* LEGE 07169. Moreover, its placement in the tree is very distant from *Gloeocapsa* sp. PCC 73106, which is a reference strain [17].

### 2.2. Bioactivity

**Figure 2:**
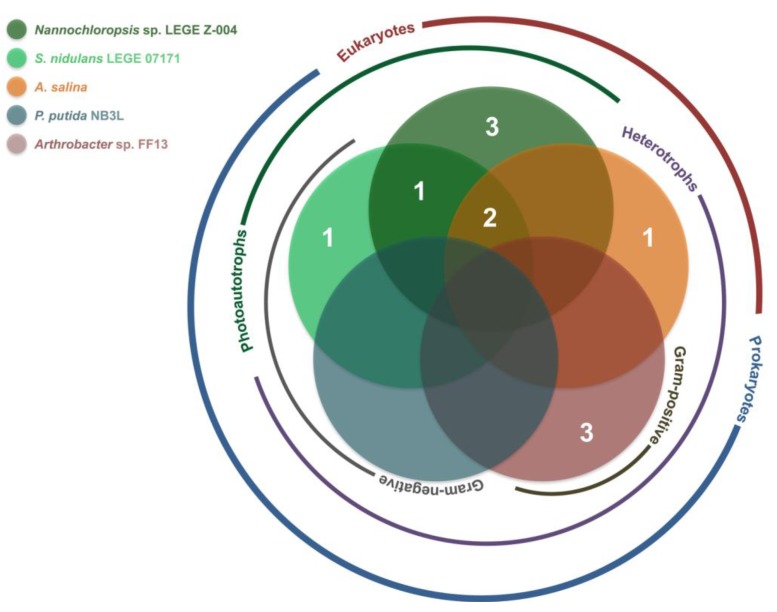
Diagram illustrating the chemoecological screening results. Circles represent each bioassay, as depicted in the legend. Numbers indicate the amount of tested strains that showed activity in one (or more, if the number lies at an intersection) bioassays (*N*_tested strains_ = 13; *N*_strains with activity_ = 11). Also depicted are general physiological characteristics of the target organisms that were taken into account in the screening design.

The results of the screening for bioactivity are summarized in Table 2 and Figure 2. Crude extracts obtained from the cyanobacterial biomass and their respective fractions were tested in a series of bioassays, using auto- and heterotrophic, prokaryotic and eukaryotic marine organisms as targets (Figure 2). These assays were selected taking into account the putative natural roles for the secondary metabolites produced by the tested marine cyanobacteria, *i.e.*, that they could be produced to elicit a response (in this case inhibitory) on a competitor (for nutrients or space) or grazer. Fractions from the majority (84.6%) of the tested strains showed activity in at least one bioassay, most of these only at 100 μg mL^−1^. The assay that employed *Nannochloropsis* sp. LEGE Z-004 as the target organism had the most hits (46.2% of the tested strains inhibited the growth of the microalga). Conversely, no inhibitory activity towards *Pseudomonas putida* NB3L was observed, up to 100 μg mL^−1^. Among the chromatographic fractions, the most polar ones (C) were the least active (corresponding to 15% of the total number of hits). Interestingly, fractions with activity in the *Arthrobacter* sp. FF13 bioassay were not active in any of the other assays.

**Table 1:** Marine cyanobacteria strains tested in this study for exploration of their bioactive compound potential and geographical distribution of the beaches from where these originated.

Taxon	Strain Code	Origin ^a^	Reference	
*Romeria* sp.	LEGE 06013	Foz do Arelho (D)	[13]	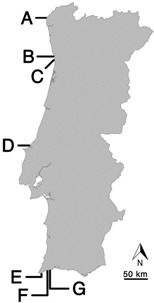
*Pseudanabaena* sp.	LEGE 06116	Martinhal (E)	[13]	
*Leptolyngbya* sp.	LEGE 06133	Moledo (A)	^b^	
*Pseudanabaena* cf. *frigida*	LEGE 06144	Burgau (F)	[13]	
Pseudanabaenaceae cyanobacterium	LEGE 06148	Moledo (A)	^b^	
*Nodosilinea nodulosa* *	LEGE 06152	Lavadores (B)	[13]	
*Nodosilinea nodulosa*	LEGE 06191	Burgau (F)	^b^	
cf. *Gloeocapsa* sp.	LEGE 06192	Burgau (F)	^b^	
*Leptolyngbya saxicola*	LEGE 07132	Luz (G)	[13]	
*Leptolyngbya* *mycoidea*	LEGE 07157	Lavadores (B)	[13]	
*Schizothrix* aff. *septentrionalis*	LEGE 07164	Moledo (A)	[13]	
*Pseudanabaena* cf. *curta*	LEGE 07169	Aguda (C)	[13]	
*Calothrix* sp.	LEGE 07177	Martinhal (E)	[13]	

^a^ all beach locations are in Portugal, capital letter under parentheses corresponds to location labels in the map of Portugal shown; ^b^ this study. * re-identified (see discussion); formerly assigned to *Leptolyngbya* cf. *halophila*.

**Table 2:** Bioactivity of crude extracts and corresponding chromatographic fractions from the selected cyanobacterial strains in a series of ecologically-relevant assays.

Strain	Active Fractions in each bioassay ^a^ (Lowest Concentration Observed) [Lethality or Inhibition, %] ^b^				
	*Artemia salina*	*Arthrobacter* sp. FF13	*Pseudomonas putida* NB3L	*Nannochloropsis* sp. LEGE Z-004	*Synechococcus nidulans* LEGE 07171
LEGE 06013	B (100 μg mL^−1^) [25.2 ± 7.3]	-	-	A; B (100 μg mL^−1^) [A: 100 ± 38.9] [B: 100 ± 12.1] *	A (100 μg mL^−1^) [54.9 ± 4.2]
LEGE 06116	-	-	-	-	-
LEGE 06133	-	A (100 μg mL^−1^) [50.3 ± 11.9]	-	-	
LEGE 06144	-	-	-	B (100 μg mL^−1^) [88.7 ± 14.3]	B (100 μg mL^−1^) [78.8 ± 13.3]
LEGE 06148	-	-	-	A (100 μg mL^−1^); B (10 μg mL^−1^) [A: 66.4 ± 18.4] [B: 93.8 ± 3.9]	-
LEGE 06152	-	B (100 μg mL^−1^) [70.5 ± 24.5]	-	-	-
LEGE 06191	-	-	-	-	-
LEGE 06192	-	-	-	-	A (100 μg mL^−1^) [67.5 ± 11.9]
LEGE 07132	B (100 μg mL^−1^) [45.9 ± 11.1]	-	-	-	-
LEGE 07157	-	-	-	B (100 μg mL^−1^) [100 ± 9.8 ] *	-
LEGE 07164	-	-	-	B; C (10 μg mL^−1^) [B: 36.5 ± 9.1] [C: 19.5 ± 7.6]	-
LEGE 07169	B (100 μg mL^−1^) [54.0 ± 10.9]	-	-	B; C (10 μg mL^−1^) [B: 38.0 ± 6.0] [C: 43.5 ± 8.1]	C (100 μg mL^−1^) [92.7 ± 1.3]
LEGE 07177	-	A (100 μg mL^−1^) [70.9 ± 24.9]	-	-	-

^a^ fractions (A, B or C) with lethality values above 20% (*A. salina* assay) or growth inhibition values above 50% of negative control (remaining assays); values under parentheses indicate the lowest test concentration for which significant (*p* < 0.05, *t*-test) activity was observed, but not necessarily above 20% for lethality or above 50% for growth inhibition; ^b^ values shown under square brackets correspond to mean ± S.D. (*n* = 3) lethality for the *A. salina* assay and growth inhibition (to control) for the remaining assays at the lowest concentration in which significant (*p* < 0.05, *t*-test) activity was observed. * average optical density in the corresponding microplate wells at the end of the experiment was inferior to that measured at the beginning of the experiment, therefore 100% is presented as maximum inhibition value.

### 2.3. Dereplication

Fractions from selected strains (with codes LEGE 06013, 06133, 06144 and 06192), belonging to different genera, were analyzed by LC-MS to allow for dereplication of known compounds. For each strain, masses in the range of 300–1500 Da did not find correspondences in the MarinLit database were observed. The mid-polar fractions from *Romeria* sp. LEGE 06013 and *Pseudanabaena* cf. *curta* LEGE 06144 contained several of such masses (examples are shown in Figure S2). Pigments and glycolipid species were successfully dereplicated in both strains. 

### 2.4. Bioassay-Guided Purification of the Constituents of *Romeria* sp. LEGE 06013

**Figure 3:**
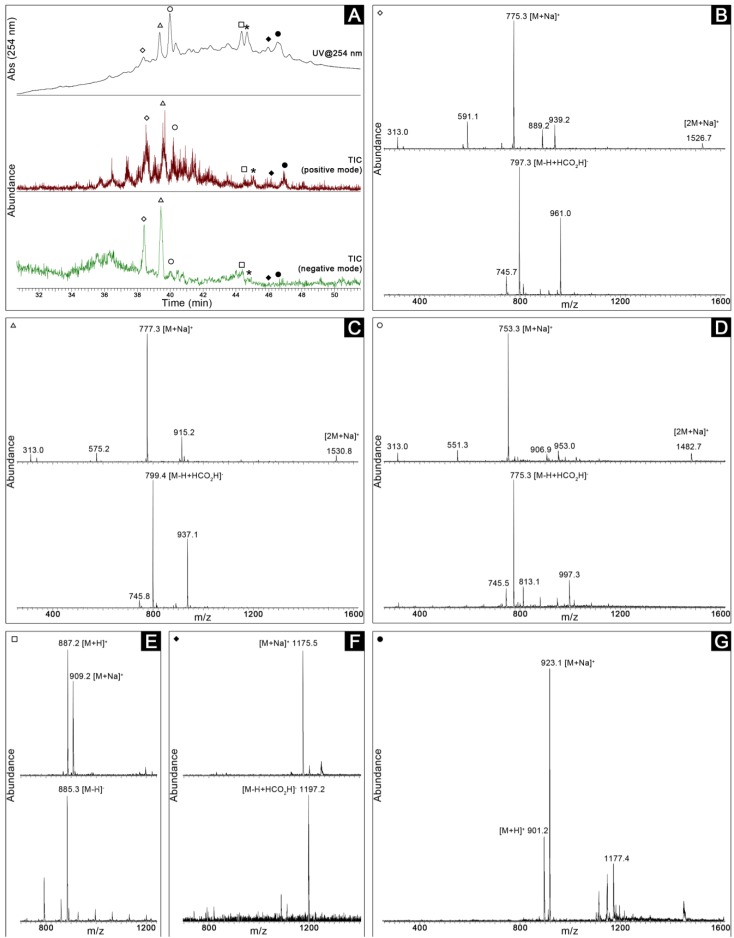
LC-MS analysis of fraction H6, obtained from *Romeria* sp. LEGE 06013. (**A**) UV (254 nm) trace and total ion chromatograms (TICs) of the fraction (ionization was only observed in the depicted time frame); the peaks labeled with an asterisk exhibit mass spectra in both positive and negative ionization modes similar to those depicted in panel E for the peak labeled with an open square, thus the corresponding compounds should be isomers. (**B**–**E**) ESI mass spectra (top: positive ionization mode, bottom: negative ionization mode) of compounds putatively identified as glycolipids (**B**–**D**: monogalactosyl diacylglycerols [acyl groups for: B linolenoyl and palmitoyl; C linoleoyl and palmitoyl; D palmitoyl]; E: digalactosyl diacylglycerol with acyl groups linolenoyl and myristoyl). (**F**) ESI mass spectra in positive (top) and negative (bottom) ionization modes for a compound with a monoisotopic mass of 1152 amu, with no correspondence in the MarinLit database (cyanobacteria). (**G**) ESI spectrum (positive ionization mode) of a compound of monoisotopic mass 900 amu that was also not found among the described marine cyanobacterial metabolites in the MarinLit database. Symbols in the top left corner of each panel correspond to chromatographic peaks labeled in panel A, and deduced ion assignments are shown.

The strain *Romeria* sp. LEGE 06013 was selected for further exploration of its active constituents, as it showed activity in several bioassays and was the fastest-growing strain among those tested. Large-scale culturing was carried out, and the resulting biomass was used to produce a crude extract. This extract was fractionated to yield nine fractions (A–I) that were tested in the bioassays for which the strain had shown the strongest activity in the chemoecological screening, *i.e.*, the assays with *Nannochloropsis* sp. LEGE Z-004 and *Synechococcus nidulans* LEGE 07171. The new fractions were tested at concentrations within the same range as the ones generated from the small-scale cultures, however, the same bioactivity pattern was not observed. In both assays, no significant differences in to the control treatment were observed in terms of cell densities. Still, in the case of the *S. nidulans* assay, it was possible to observe an abnormally large cell size (when compared to control) in cells exposed to fraction H (not shown). ^1^H NMR data (Figure S3) for this fraction indicated that its main constituents were glycolipid species, based on the characteristic resonances in the δ 4.5–3.5 and δ 5.4–5.2 regions. The fraction was subject to reversed-phase chromatography, yielding eight fractions that were again tested for activity towards *S. nidulans* LEGE 07171. The same abnormal shape pattern was observed in cells exposed to fraction H6 (Figure S4) and also H5 (albeit to a much lesser extent). LC-MS analyses of the main constituents of these fractions showed that several glycolipid species were present in the fractions. Fraction H6, in particular, appears to contain three monogalactosyl diacylglycerol species and one digalactosyl diacylglycerol (Figure 3), all of which reported in the literature as being produced by cyanobacteria [18,19,20]. Two compounds with masses for which no correspondence was found among cyanobacteria in the database MarinLit were also detected in this fraction (Figure 3).

## 3. Discussion

In the present study, all but two of the tested cyanobacterial strains showed important activity in at least one of the target bioassays, highlighting their high potential for discovery of new chemical entities. Most fractions were active only at a relatively high concentration (100 μg mL^−1^), unlikely to occur in the natural medium. It should be noted, however, that the main components of these very complex fractions are pigments and primary metabolites, as the cyanobacteria were cultured in near-optimal growth conditions. The high hit rate observed in the screening may have been the result of using a multiple bioassay strategy, in particular, with ecologically relevant assays. While *Nannochloropsis* sp. LEGE Z-004 was the most susceptible target organism to the organic constituents of the cyanobacteria, if the screening had been conducted with this single assay, the number of potentially bioactive-compound producing strains would have been underestimated by nearly half. On the other hand, if we had employed only the assays with *Nannochloropsis* sp. and the Gram-positive *Arthrobacter* sp. FF13, we would have identified ~80% of the bioactive strains. Still, the other two assays that resulted in activity (those with *S.* *nidulans* and *Artemia salina* as target organisms) each allowed the identification of one active strain that did not show activity in any of the other assays. This highlights the importance of bioassay choice to uncover new chemical entities through bioassay-guided approaches. Also of interest is the lack of hits in the *P. putida* bioassay, while the other Gram-negative assay organism, *S. nidulans*, was affected by several of the tested strains. This suggests that active metabolites from these latter may possess a cell-wall-independent mode of action and, because all but one of the strains that inhibited the growth of the cyanobacterium also inhibited the growth of *Nannochloropsis* sp., the photosynthetic apparatus function may be the biochemical target of these compounds. In fact, allelopathic interactions, in particular those targeting photosynthetic machinery, are a known feature of cyanobacteria [21]. Nevertheless, the lack of sensitivity of *P. putida* to the cyanobacterial extracts could also be explained by the well-known catabolic ability exhibited by members of this species (e.g., [22]).

One other important explanation for the high hit rate in this screening effort may reside in the biodiversity of the tested cyanobacterial isolates. Although the strains herein used, according to the botanical system of classification, were initially assigned for the most part to two different genera belonging to the oscillatorialean family Pseudanabaenaceae (*i.e.*, Pseudanabaena and Leptolyngbya), it became evident from their distribution in the 16S rRNA tree that their morphological identifications lacked phylogenetic support. These morphologically simple, thin, filamentous cyanobacteria have been previously described to possess a greatly underestimated biodiversity [23,24], which cannot be resolved by traditional systems of classification, which are primarily based on microscopic characterization. Furthermore, even the traditional orders (or comparable subsections in Bergey’s manual) of cyanobacteria [25,26,27], or several hitherto well recognized genera (see, for example, Honda *et al.* [16]; Casamatta *et al.* [24]; Marquardt and Palinska [28]), are known to be polyphyletic, with some unicellular or colonial forms being placed within clades of filamentous morphotypes [16,26]. This has led to the widespread recognition of the need for a thorough taxonomic revision of these organisms. Therefore, several taxonomic studies have proposed new genera for distinct phylogenetic groups of cyanobacteria previously assigned to a “traditional” taxon, based primarily on molecular data (e.g., Abed *et al.* [14], Bohunicka *et al.* [29], Siegesmund *et al.* [30]). Indeed, this is the case for strain UTEX 2910 which was early classified as the new species *Leptolyngbya nodulosa* [31] and more recently re-classified as *Nodosilinea nodulosa* [32]. Some of the strains used in this study, previously assumed as *Leptolyngbya* spp. and an unidentified Pseudanabaenaceae species, fall in the clade of this new genus (Figure 1), members of which characteristically exhibit the unique ability to form nodules along the length of the filament when grown at low light levels [32]. In fact, the strains LEGE 06152 and 06191, which are phylogenetically very close to the type species strain (UTEX 2910), possess this unique feature under our laboratory conditions (see Brito *et al*. [13], Table S1 and Figure S1). Despite being the most closely related strains used in this study, these two isolates did not show the same bioactivity potential because, unlike LEGE 06152, no active fractions were detected for LEGE 06191 (Table 2). This may be due to lower production levels of the same bioactive compound(s) than strain LEGE 06152, or, as is known for other closely related strains of cyanobacteria, one produces a specific secondary metabolite while the other has lost this ability over the course of its evolution [33,34]. Also, the closely-related phylotypes LEGE 06148 and LEGE 06133 did not exhibit the same pattern of bioactivity. Thus, we can infer that the phylogenetic relationship among strains is not a suitable criterion *per se* for the selection of particular taxa (*i.e.*, phylotypes) when aiming to search for novel secondary metabolites. On the other hand, strains assigned to the same genus, and thus presumed to be closely related, may prove to be phylogenetically distant taxa, as was the case for the *Pseudanabaena* and *Leptolyngbya* strains selected from our culture collection. These kinds of apparent incongruities may originate from either misidentifications of the strains or as an inherent consequence of problems in the taxonomy of the Cyanobacteria, which is still under revision. Hence, each cyanobacterial isolate can and should be perceived as having the potential to represent a source of unique chemodiversity.

Previous studies from our group employing laboratory cultures of cyanobacteria isolated from Portuguese waters demonstrated the biological activity of strains from estuarine environments [35] as well as that of cultured picoplanktonic marine strains [36]. The present data is, unfortunately, not directly comparable to these studies as the concentrations used in the afore-mentioned reports were considerably higher (mg mL^−1^ range). The current report constitutes, to our knowledge, the first study to evaluate the bioactivity of extracts from filamentous cyanobacteria from the Portuguese coast. In fact, there are still only a few published studies with the aim of exploring the potential of cultured marine cyanobacteria from Europe for new biologically active natural products (e.g., [37,38]). It is thus plausible that considerable chemical diversity remains to be discovered from these organisms, in particular from those that do not reach high densities in their natural habitats and thus require significant culture effort in order to explore for their bioactive constituents. The marine strains used in this study fall in this latter category because the Portuguese coast is exposed to high wave energy [39] and high tidal amplitudes [40], conditions which do not allow for the development of mat- or tuft-forming cyanobacteria. The high bioprospecting potential of these strains is evidenced by the LC-MS analyses performed in which several components of selected fractions had no counterpart in the MarinLit database. In this regard, cyanobacterial culturing efforts assume great relevance, even as culture-independent secondary metabolite discovery efforts become more widely envisioned [41].

This screening approach allowed the identification of several cyanobacterial strains as particularly promising for subsequent chemical exploration. In the case of *Romeria* sp. LEGE 06013, the inhibitory activities observed in the screening assay towards photosynthetic and non-photosynthetic eukaryotes as well as a cyanobacterium are of particular interest. These activities were, however, difficult to follow after a new extraction of *Romeria* sp. LEGE 06103 biomass after large-scale culturing. One explanation for this lies in the fact that culture conditions were not completely identical (vessel size, time) which may have yielded *Romeria* sp. cells in different physiological states and, thus, different bioactivities (see, for example [42]). The number of fractions obtained from the two crude extracts (three in the small-scale and nine in the large-scale approach) can also lead to such differences, for example due to the potential disruption of synergisms. These factors should be taken into account when considering a similar strategy for natural product screening and isolation. Nevertheless, we believe our strategy is particularly valuable as a means of selecting strains for downstream work. The observed effects of the purified fraction (H6) from *Romeria* sp. LEGE 06013, on *S. nidulans* LEGE 07171 cells could have been caused by the high concentration of glycolipids, which are membrane constituents in cyanobacteria and are known to cause similar effects in cyanobacteria [19]. Another possibility is that some of the minor constituents of the purified fraction are responsible for the observed effects. Nevertheless, purification of these latter minor compounds will constitute further work and should be of interest, as their masses do not correspond to known marine cyanobacterial metabolites present in the MarinLit database, and because this genus has, to our knowledge, never been studied in terms of secondary metabolite production.

Future explorations of *Pseudanabaena* cf. *curta* LEGE 07169, and the Pseudanabaenaceae cyanobacterium LEGE 06148 should also be of interest, as these strains yielded the most potent fractions (active at 10 μg mL^−1^) in the present study. To our knowledge, only the odorous compound 2-methyl-isoborneol [43] and the toxin microcystin-LR (detected indirectly by ELISA) [44], have been reported from the genus *Pseudanabaena*. Fractions from *Schizothrix* aff. *septentrionalis* LEGE 07164 were also active at the lowest tested concentration. In contrast with the genus *Pseudanabaena*, a large number of secondary metabolites (6% of total from cyanobacteria) is attributable to the genus *Schizothrix* [45]. However, most of these records correspond to metabolites isolated from mixed assemblages of *Lyngbya* *majuscula* (now *Moorea* spp. [2]) with *Schizothrix* spp. (e.g., [46,47,48]), and thus it remains uncertain which was the metabolite-producing strain. Still, geosmin [49], schizotrin A [50] and gallinamide A [51] have been isolated from *Schizothrix* spp. that were not part of a consortium, and thus there is at least some degree of secondary metabolism confirmed within the genus. Nevertheless, and as stressed above, such taxonomic generalizations and/or deductions need to be exercised with great caution.

## 4. Experimental Section

### 4.1. Cyanobacterial Cultures

Thirteen cyanobacterial strains from our culture collection, isolated as described in Brito *et al.* [13] from intertidal rocky beaches along the Portuguese continental coast, were selected for this study (Table 1 and Figure 1). Four of these strains, not previously described in Brito *et al.* [13], were morphologically characterized and identified according to the botanical system of classification [52,53] (see Table S1 and Figure S1 for details). Phenotypic and genotypic data from each strain were then compared, and the identifications re-evaluated.

The cyanobacteria were chosen so as to maximize diversity among the filamentous forms present in the LEGE culture collection but also considered those with growth rates suitable for downstream work. One colonial unicellular strain was also included. Uni-cycanobacterial cultures of each of these strains were maintained in either Z8 medium [54] supplemented with 25 g L^−1^ NaCl or MN medium [55], at 25 °C and under a 14:10 h light (~25 μmol m^−2^ s^−1^ photon irradiance):dark cycle in 2 L Erlenmeyer flasks. Both media were supplemented with 10 μg mL^−1^ vitamin B_12_. After 30–45 days of growth, the cyanobacterial biomass was harvested, rinsed with deionized water and lyophilized.

### 4.2. 16S rRNA Gene Amplification, Cloning and Sequencing

Total genomic DNA (gDNA) was isolated from fresh biomass samples, harvested from cultures of LEGE 06133, LEGE 06191, LEGE 06192 and LEGE 06148 (strains that had not been genetically characterized), using a commercial kit (PureLink™ Genomic DNA Mini Kit, Invitrogen, Carlsbad, USA). Primers CYA 359F [56] and 1494Rc [57] were used to PCR-amplify a portion of the 16S rRNA gene. Amplicons were purified from agarose gel slices (Cut & Spin columns, GRiSP, Porto, Portugal), cloned into pGEM-T^®^ Easy vector (Promega, Madison, WI, USA), and then transformed into OneShot^®^ TOP10 cells (Invitrogen). Plasmid DNA was isolated using GenElute™ Plasmid Miniprep Kit (Sigma-Aldrich, St. Louis, MO, USA) and sequenced (Macrogen Inc., Seoul, Korea) using M13 primers. The sequences were checked for quality and deposited in GenBank under the accession numbers KC249949-KC249952.

### 4.3. Phylogenetic Analysis

A 16S rRNA gene phylogeny was constructed to gain insight into the cyanobacterial diversity among the tested isolates. The sequences from each of the LEGE cyanobacterial isolates were queried using BLASTn, and the corresponding top-scoring sequences were used in the phylogenetic analysis. Moreover, to obtain a reliable and taxonomically relevant phylogenetic tree structure, several sequences were included from Bergey’s reference strains or from their close relatives (*i.e.*, strains known to belong to a certain cluster or sub-cluster from which the 16S rRNA sequence of the reference strain is not publicly available) [17]. One type species from a recently proposed new genus, *Nodosilinea* [32], was also considered in the analysis for taxonomic identification and clarification.

A multiple sequence alignment was performed in MEGA version 5 [58] using the ClustalW algorithm. jModelTest 2 [59] was then used to evaluate which model of nucleotide substitution best-fit the dataset. As a measure of the goodness of fit of these models, the corrected Akaike’s Information Criterion (AICc) was employed. This allowed us to select the GTR + Gamma + Proportion Invariant (GTR + G + I) model of evolution. MrBayes version 3.1.2 [60] was then used for Bayesian inference (BI), to estimate the posterior probability distribution using the Bayesian Markov chain Monte Carlo (MCMC) method. The phylogenetic tree reconstruction was performed using a random starting tree, while one cold and seven incrementally heated chains (temperature set 0.2) were run for 10^7^ generations, with a tree sampling frequency of 100. This was made in two independent runs, and the resulting consensus phylogeny was built from the last 75% of trees.

The same alignment was also used to generate a Maximum-likelihood (ML) bootstrap tree (1000 replicates), using MEGA 5 software. The tree was reconstructed by using the same parameters as the individual Bayesian inferred tree. Due to the existence of several sequences considerably smaller on the alignment, positions containing more than 10% of ambiguities (alignment gaps and missing data) were deleted. The topologies retrieved from the different analyses were then evaluated using TreePuzzle 5.2 [61]. All of the test comparisons (one and two-sided Kishino-Hasegawa test, Shimodaira-Hasegawa test, Expected Likelihood Weights) indicated that the Bayesian tree yielded the best topology for our dataset. The branch support values derived from the ML analysis were then compared using TreeGraph 2 [62].

### 4.4. Extraction and Fractionation

The freeze-dried biomass from each cyanobacterial strain (>100 mg) was repeatedly extracted with warm (<40 °C) CH_2_Cl_2_:MeOH (2:1), filtered through Whatman No 1 paper and the solvents removed under reduced pressure. A portion of this crude extract was then fractionated through a normal phase (SiOH) SPE cartridge (Strata SI-1, Phenomenex), using a polarity gradient from 100% hexanes to 100% EtOAc to 100% MeOH. Three fractions were obtained (termed A, B and C in order of increasing polarity), which were dried and stored at −20 °C. All solvents used were ACS grade.

### 4.5. Bioassays

The crude extracts and fractions were dissolved in DMSO (10 and 1 mg mL^−1^) and tested to a panel of ecologically-relevant bioassays. These were performed in 96-well microplates with the extracts or fractions being tested at concentrations of 100 and 10 μg mL^−1^ in a total volume of 200 μL per well. DMSO at 1% (v/v) in the culture medium was used as a negative control.

Further details of each bioassay, including positive controls, are given below:

(a) *Artemia salina* (brine shrimp) toxicity assay. The assay was conducted as previously reported [35]. Mortality rates were determined at 24 and 48 h following exposure and a 0.4 mg mL^−1^ potassium dichromate solution in DMSO was used as a positive control (4 μg mL^−1^ final concentration in each well).
(b) *Arthrobacter* sp. FF13. This strain was isolated from *Fucus spiralis* in Porto, Portugal (41°09′N; 8°40′W). Liquid cultures in M607 medium [63] were grown in the dark at 25 °C with shaking to exponential phase and diluted to 0.1 OD (750 nm) in each of the assay wells in M607 medium. A mixture of penicillin (50 units mL^−1^), streptomycin (50 μg mL^−1^) and neomycin (100 μg mL^−1^) was used as a positive control (values are final concentrations). Plates were incubated under the conditions described for the batch cultures. Growth of the treatments and controls was evaluated by OD measurements at 750 nm after 48 h.
(c) *Pseudomonas putida* NB3L was isolated from a sponge belonging to the “*Cliona viridis* complex” at 4 to 5 m depth in Parque Natural da Berlenga, Portugal. The assay was performed as described above for *Arthrobacter* sp. FF13, however, in this case, a simple medium of filtered (0.22 μm) seawater supplemented with peptone (5 g L^−1^) and yeast extract (1 g L^−1^) was used.
(d) A marine *Nannochloropsis* sp. strain (LEGE Z-004) was used to study the phycotoxic or potential allelopathic properties of the extracts. The microalgae were grown in batch culture in Z8 medium supplemented with 25 g L^−1^ NaCl under the same light and temperature conditions as described above for the cyanobacterial strains. The strain was inoculated to ~0.1 OD (750 nm) in each of the microplate wells. Potassium dichromate served as positive control as described above for the *Artemia salina* bioassay. The 96-well microplate was incubated under the same conditions as the batch cultures and OD at 750 nm was used to measure growth after 72 h.
(e) The marine cyanobacterium *Synechococcus nidulans* LEGE 07171 was used as a photosynthetic prokaryotic target from a ubiquitous genus. The bioassay employing this strain was performed as described for *Nannochloropsis* sp. LEGE Z-004, however, the positive control was the antibiotic mixture mentioned above for the other prokaryotic targets. Growth was estimated by OD measurements (750 nm) after 120 h.

We considered that an extract or fraction was active if a minimum of 20% mortality in the *A. salina* bioassay and of 50% growth inhibition to control in the remaining assays was observed for that particular sample, taken that these differences were significant at a 95% confidence level (Student’s *t*-test).

### 4.6. LC-MS Analyszzes and Dereplication

Fractions from selected active strains (*Romeria* sp. LEGE 06013 and *Pseudanabaena* cf. *frigida* LEGE 06144) were profiled by LC-MS, with the objective of dereplicating known compounds that could be present in the fractions and identifying mass signatures that could correspond to unknown metabolites. Samples were dissolved in MeOH at a concentration of 0.5–1.2 mg mL^−1^. The analyses were performed on a Thermo LCQ Fleet Ion Trap LC/MS^n^ system (Thermo Scientific, Waltham, MA, USA) equipped with a C18 Hypersil Gold column 100 × 4.6 mm i.d., 5 μm (Thermo Scientific). A gradient from 20% aq. MeOH to 100% MeOH for 60 min was used in the LC separation. Positive and negative ions were analyzed using electrospray ionization (ESI) and MS data were acquired in full-scan mode (200–2000 *m/z*). The ESI sheath gas was operated at 80 (unitless), the auxiliary gas at 20 (unitless) and the heated capillary temperature was set at 350 °C. All solvents used were LC-MS grade. The mass spectra originating from each of the observable chromatographic peaks (200–400 nm) were then annotated, and compound masses deduced from at least two molecular species (e.g., protonated and sodium adduct) were queried in the MarinLit database (University of Canterbury, New Zealand).

### 4.7. Bioassay-Guided Purification of the Constituents of *Romeria* sp. LEGE 06013

Large-scale culturing of *Romeria* sp. LEGE 06013 was carried out in 6 L round bottom flasks, under the same growth conditions described in Section 4.1, to afford 16.7 g of freeze-dried biomass. A crude extract (2.9 g) was prepared from this material as described in Section 4.4 Normal-phase (silica gel 60, 0.015–0.040 mm, Merck KGaA, Damstadt, Germany) Vacuum Liquid Chromatography (VLC), using a gradient from 100% *n*-hexane to 100% EtOAc to 100% MeOH was used to fractionate a portion (2.8 g) of the crude extract, to yield nine fractions (A–I). These were tested in duplicate in the *Nannochloropsis* sp. LEGE Z-004 and *S. nidulans* LEGE 07171 assays as described above. Further purification of a portion of the active fraction H (300 mg) was achieved by reversed-phase column chromatography on an SPE cartridge (Strata C_18_, 10 g, Phenomenex), yielding eight fractions (H0–H7). These were tested in the *S. nidulans* assay at 30 μg mL^−1^ in duplicate. Fractions H5 (99 mg) and H6 (35 mg) were analyzed by LC-MS as described in Section 4.5, except for the use of acidified (0.1 formic acid) mobile phase. The ^1^H NMR spectrum of fraction H was recorded in a Varian Inova spectrometer operating at 500 MHz. A putative identification of the compounds was carried out using MarinLit (see Section 4.5) as well as searches in published literature on cyanobacterial glycolipids [18,19,20].

## 5. Conclusions

This study employed a non-targeted screening strategy with a strong emphasis on ecologically-relevant bioassays, together with a selection of novel and diverse cyanobacterial isolates. As predicted, this approach led to a high hit rate of biological activity, which in turn, allowed us to perform an LC-MS based selection of promising cyanobacterial strains for further chemical exploration. We studied with more detail one of these strains regarding its bioactive components, which appear to be one or several glycolipids, however unknown components, which may be of interest, were also detected. Future structural characterization of compounds isolated from these strains will allow us to better evaluate the value of this type of strategy for bioactive compound discovery from marine cyanobacteria. However, a general finding of this study is that a less-targeted screening strategy that employs more bioassays may result in an increased pool of selected strains for further studies.

## Supplementary Files

Supplementary File 1 Supplementary Information (PDF, 731 KB)
