# Supplementary material for: Chemoecological Screening Reveals High Bioactivity in Diverse Culturable Portuguese Marine Cyanobacteria
Source: Mar Drugs. 2013 Apr 22;11(4):1316–35. doi: 10.3390/md11041316 (PMC3705407; doi:10.3390/md11041316)
Supplement: Supplementary File 1 — Supplementary Information (PDF, 731 KB) [file marinedrugs-11-01316-s001.pdf]

Supplementary Information

Table S1. Origin and morphological description of marine cyanobacterial strains selected from LEGE culture collection (the other strains used in this study have been characterized in Brito, *et al.* [1]).

Taxon [a]	LEGE strain code	Sampling site	Habitat and ecological description	Brief morphological characterization [b]	Microphotographs
Leptolyngbya saxicola	06133	Lavadores beach	lower intertidal zone, tide pool, submerged stone, epilithic	straight or broadly curved filaments; w/tight sheath; not constricted at the cell walls; no visible nodule-like structures; cells much longer than wide: 5.2 ± 1.0 × 0.9 ± 0.1 μm; rounded apical cells	Figure S1
unidentified Pseudanabaenaceae	06148	Luz beach	middle intertidal zone, wave-exposed rock surface, epilithic	wavy or spirally coiled filaments; occasionally, filaments densely packed together forming a disordered structure, deeply constricted at cross-walls, sheath up to 0.5 μm, cells mainly wider than long: 0.9 ± 0.2 × 1.4 ± 0.2 μm, rounded apical cells	
Nodosilinea nodulosa	06191	Burgau beach	seawater, surf zone, plankton [c]	slightly straight or broadly curved filaments; w/ tight sheath; constricted at cross-walls; w/visible "chromatoplasm" (=parietal thylakoids); forms distinctive nodule-like structures; cells mostly isodiametric: 1.5 ± 0.4 × 1.2 ± 0.1 μm	
cf. *Gloeocapsa*	06192	Burgau beach	seawater, surf zone, plankton [c]	unicellular, colonial; greenish-brown; cells and sub-colonies surrounded by wide gelatinous sheaths, forming mucilaginous colonies; Ø cells: 1.5 ± 0.3 μm; Ø sub-colonies: up to 11 μm	

[a] Taxa were identified according to standard botanical classification system [2,3] or reclassified in agreement to recent taxonomic studies [4];

[b] cell size: length × width (for filamentous forms); all values represent mean ± SD (n = 20);

[c] Note: although present in plankton, the strains should not be assumed as planktonic.

Figure S1. Photomicrographs of the isolates depicted in Table S1. Scale bar = 20 μm (1000×) or 50 μm (400×).

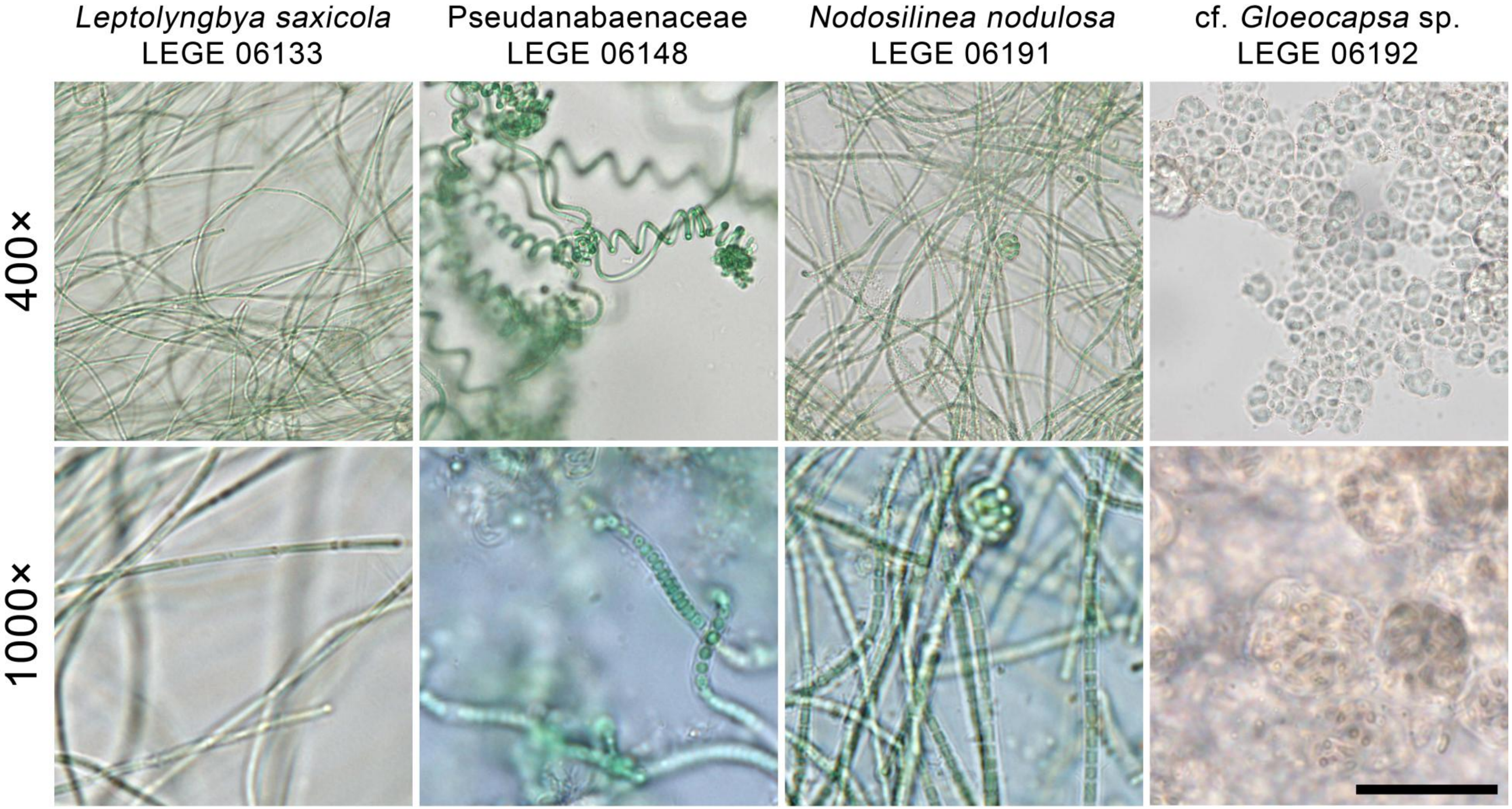

Figure S2. LC-MS analyses of fractions "B" (mid-polarity) from strains *Romeria* sp. LEGE 06013 and *Pseudanabaena* cf. *frigida* LEGE 06144, illustrating the presence of several unknown metabolites. Top panel corresponds to the LC trace, with selected chromatographic peaks marked with a geometrical figure and respective retention time. Remaining panels correspond to the positive ion mode mass spectrum for each of the selected chromatographic peaks. Mass over charge values for $[M + H]^+$ and $[M + Na]^+$ species are denoted in the mass spectra and were used to derive the mass range queried for each metabolite in MarinLit, which is also indicated in each panel.

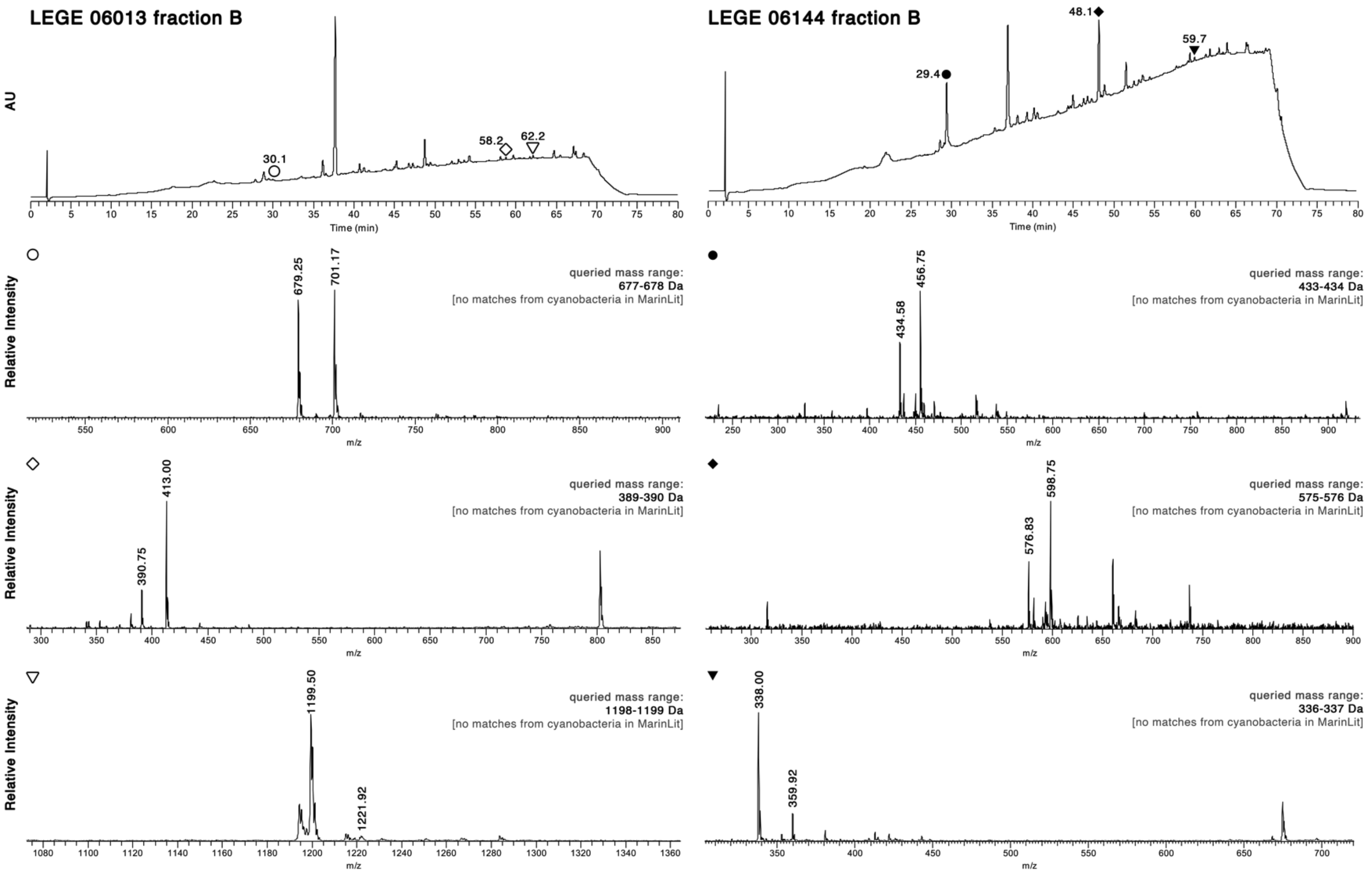

Figure S3. ^{1}H NMR spectrum ($CDCl_3$, 500 MHz) of VLC fraction H, obtained from the biomass of *Romeria* sp. LEGE 06013.

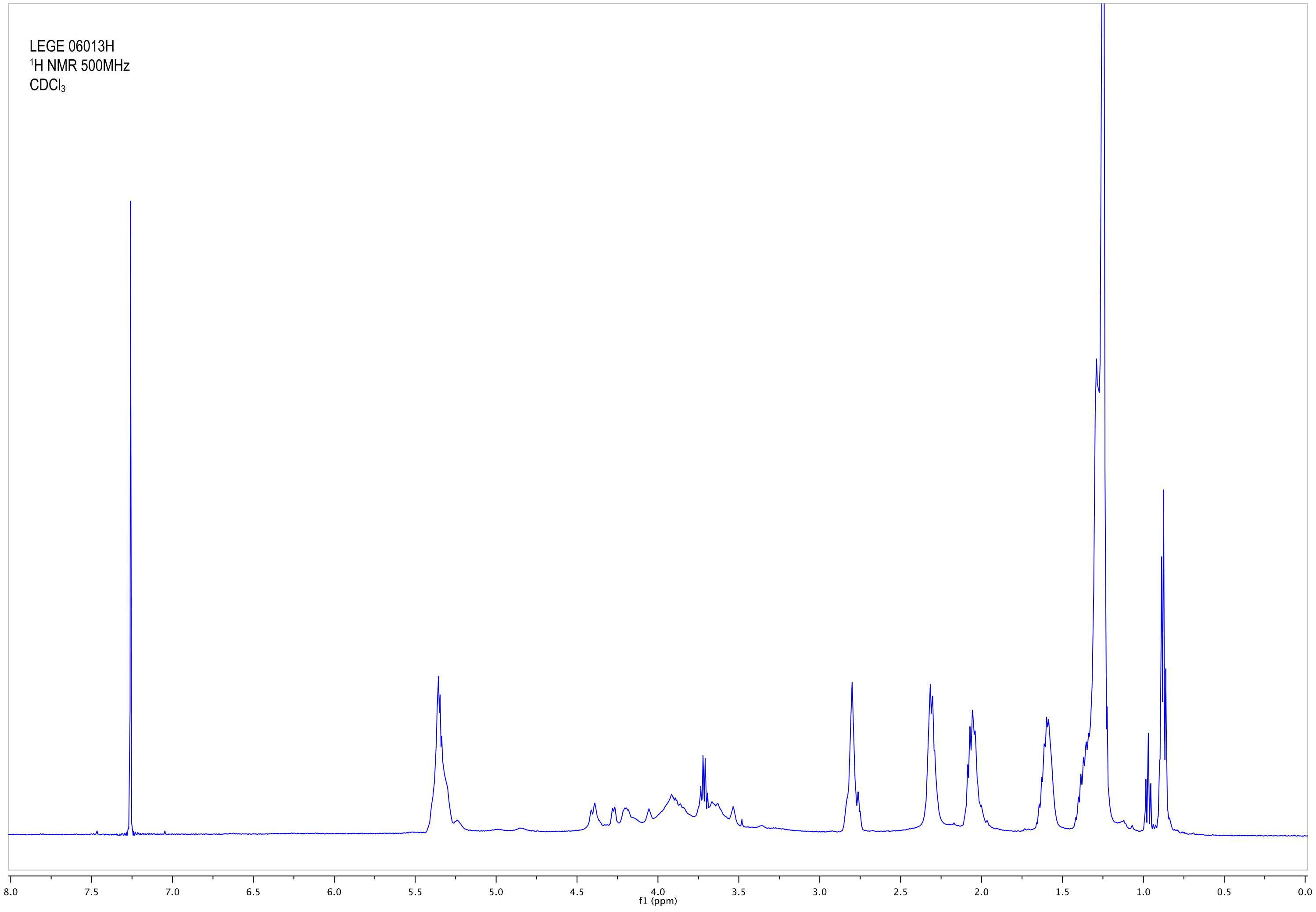

Figure S4. Abnormal cell shape in *Synechococcus nidulans* LEGE 07171 following exposure to fraction H6 from *Romeria* sp. LEGE 06013. Photomicrographs obtained from different microplate wells are shown. Left panel: *S. nidulans* LEGE 07171 cells, 24 h after being inoculated in 1% DSMO in Z8 medium (supplemented with 25 g L^{-1} NaCl) (control treatment). Right panel: *S. nidulans* LEGE 07171 cells, 24 h after being inoculated in Z8 medium (modified with NaCl as described for the control treatment) containing 1% of a 3 mg mL^{-1} solution of fraction H6 in DMSO. Arrows indicate examples of cells with abnormal shape (size). The photomicrographs were acquired using an inverted microscope (DMIL, Leica Microsystems, Wetzlar, Germany) equipped with an ICCA camera and Qwin Colour software (Leica Microsystems).

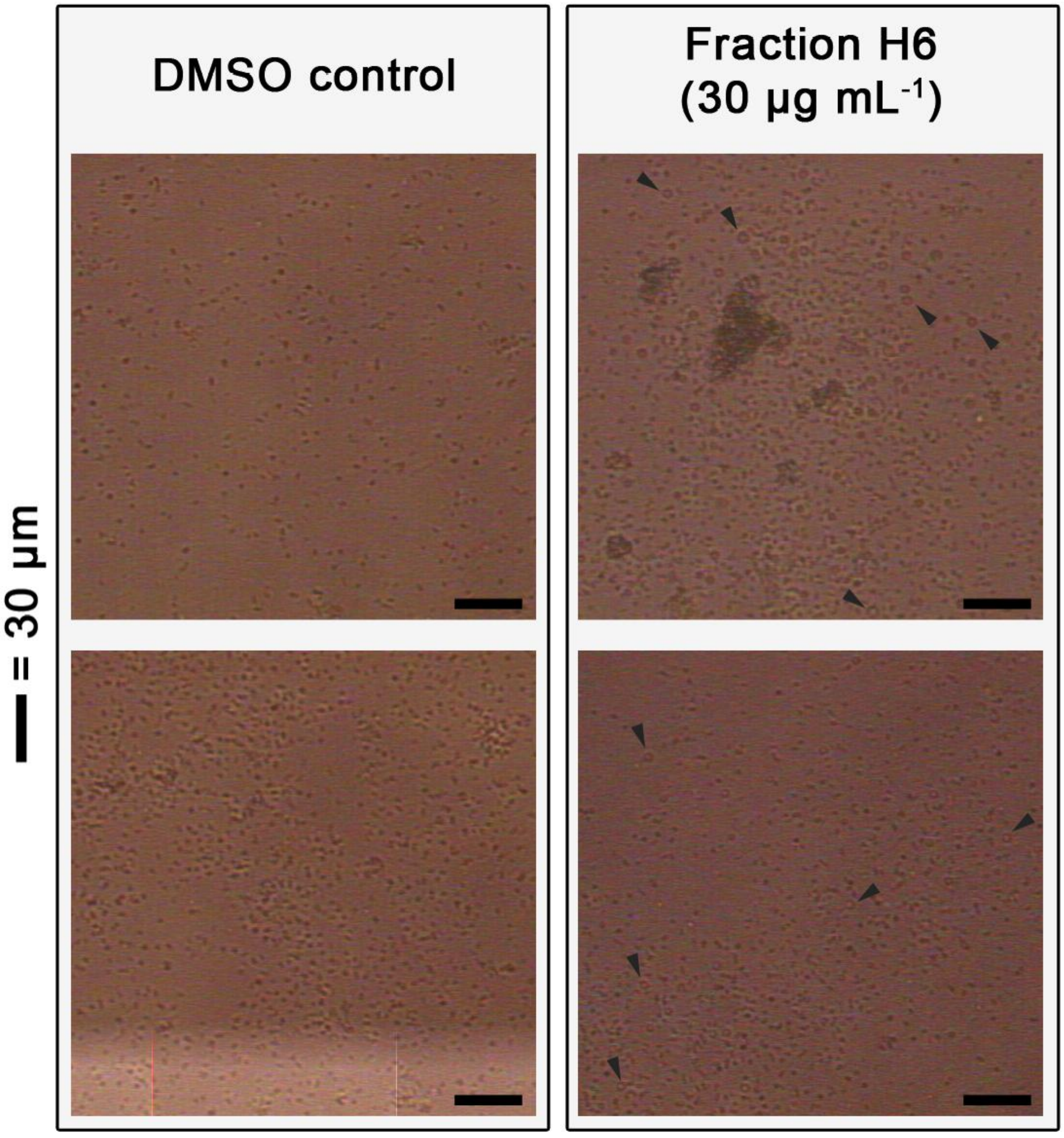

References

1. Brito, A.; Ramos, V.; Seabra, R.; Santos, A.; Santos, C.L.; Lopo, M.; Ferreira, S.; Martins, A.; Mota, R.; Frazão, B.; *et al.* Culture-dependent characterization of cyanobacterial diversity in the intertidal zones of the Portuguese coast: A polyphasic study. *Syst. Appl. Microbiol.* **2012**, *35*, 110–119.
2. Komárek, J.; Anagnostidis, K. Cyanoprokaryota, Part 1: Chroococcales. In *Süsswasserflora von Mitteleuropa*; Ettl, H., Gärtner, G., Heynig, H., Mollenhauer, D., Eds.; Spektrum Akademischer Verlag: Heidelberg, Germany, 1998; Volume 19/1, p. 548.
3. Komárek, J.; Anagnostidis, K. Cyanoprokaryota, Part 2: Oscillatoriales. In *Süsswasserflora von Mitteleuropa*; Büdel, B., Gärtner G., Krienitz L., Schagerl M., Eds.; Elsevier: Munich, Germany, 2005; Volume 19/2, p. 759.
4. Perkerson, R.B.; Johansen, J.R.; Kovacik, L.; Brand, J.; Kastovsky, J.; Casamatta, D.A. A unique Pseudanabaenalean (Cyanobacteria) genus *Nodosilinea* gen. nov. based on morphological and molecular data. *J. Phycol.* **2011**, *47*, 1397–1412.

© 2013 by the authors; licensee MDPI, Basel, Switzerland. This article is an open access article distributed under the terms and conditions of the Creative Commons Attribution license (http://creativecommons.org/licenses/by/3.0/).
